# Synovial Fluid C-reactive Protein Clinical Decision Limit and Diagnostic Accuracy for Periprosthetic Joint Infection

**DOI:** 10.7759/cureus.52749

**Published:** 2024-01-22

**Authors:** John L Miamidian, Krista Toler, Alex McLaren, Carl Deirmengian

**Affiliations:** 1 Department of Diagnostics Research and Development, Zimmer Biomet, Warsaw, USA; 2 Orthopaedic Surgery, University of Arizona College of Medicine - Phoenix, Phoenix, USA; 3 Orthopaedic Surgery, Rothman Orthopaedic Institute, Philadelphia, USA; 4 Orthopaedic Surgery, Thomas Jefferson University, Philadelphia, USA

**Keywords:** c-reactive protein (crp), hip and knee arthroplasty, diagnostic performance, periprosthetic joint infection, clinical laboratory, synovial fluid analysis

## Abstract

Introduction

C-reactive protein (CRP) has long served as a prototypical biomarker for periprosthetic joint infection (PJI). Recently, synovial fluid (SF)-CRP has garnered interest as a diagnostic tool, with several studies demonstrating its diagnostic superiority over serum CRP for the diagnosis of PJI. Although previous studies have identified diagnostic thresholds for SF-CRP, they have been limited in scope and employed various CRP assays without formal validation for PJI diagnosis. This study aimed to conduct a formal single clinical laboratory validation to determine the optimal clinical decision limit of SF-CRP for the diagnosis of PJI.

Methods

A retrospective analysis of prospectively collected data was performed using receiver operating characteristic (ROC) and area under the curve (AUC) analyses. Synovial fluid samples from hip and knee arthroplasties, received from over 2,600 institutions, underwent clinical testing for PJI at a single clinical laboratory (CD Laboratories, Zimmer Biomet, Towson, MD) between 2017 and 2022. Samples were assayed for SF-CRP, alpha-defensin, white blood cell count, neutrophil percentage, and microbiological culture. After applying selection criteria, the samples were classified with the 2018 ICM PJI scoring system as "infected," "not infected," or "inconclusive." Data were divided into training and validation sets. The Youden Index was employed to optimize the clinical decision limit.

Results

A total of 96,061 samples formed the training (n = 67,242) and validation (n = 28,819) datasets. Analysis of the biomarker median values, culture positivity, anatomic distribution, and days from aspiration to testing revealed nearly identical specimen characteristics in both the training set and validation set. SF-CRP demonstrated an AUC of 0.929 (95% confidence interval (CI): 0.926-0.932) in the training set, with an optimal SF-CRP clinical decision limit for PJI diagnosis of 4.45 mg/L. Applying this cutoff to the validation dataset yielded a sensitivity of 86.1% (95% CI: 85.0-87.1%) and specificity of 87.1% (95% CI: 86.7-87.5%). No statistically significant difference in diagnostic performance was observed between the validation and training sets.

Conclusion

This study represents the largest single clinical laboratory evaluation of an SF-CRP assay for PJI diagnosis. The optimal CRP cutoff (4.45 mg/L) for PJI, which yielded a sensitivity of 86.1% and a specificity of 87.1%, is specific to the assay methodology and laboratory performing the assay. We propose that an SF-CRP test with a laboratory-validated optimal clinical decision limit for PJI may be preferable, in a clinical diagnostic setting, to serum CRP tests that do not have laboratory-validated clinical decision limits for PJI.

## Introduction

C-reactive protein (CRP) is a general inflammatory biomarker for inflammation utilized in several medical subdisciplines. Despite the emergence of more accurate biomarkers for periprosthetic joint infection (PJI), the serum CRP remains as a core component of all authoritative definitions of PJI of the hip and knee [1-6]. While the serum CRP is generally considered a non-specific systemic biomarker for inflammation, numerous recent studies indicate that the synovial fluid (SF)-CRP may demonstrate equivalent or superior performance than serum CRP for the diagnosis of PJI [7-12]. These studies have demonstrated the potential for SF-CRP to serve as an important feature of PJI definitions. 

The appropriate clinical laboratory measurement of SF-CRP requires both the optimization and validation of existing serum immunoassays. The diagnostic performance of an assay is dependent on the clinical decision limit, otherwise known as the cut-off or threshold for positivity. The process of assay validation is specific to the assay method, the laboratory performing the assay, and the disease that is intended to be diagnosed. SF-CRP assays are generally obtained from a specialty laboratory in the form of a lab-developed test (LDT), and there is no widely available or FDA-cleared SF-CRP assay. Optimal clinical decision limits for the SF-CRP have been demonstrated to vary depending on the assay format [2,7,8,10-24], supporting the need for a clinical validation of an assay-specific clinical decision limit by each laboratory offering the test for the diagnosis of PJI. Although previous studies in the literature have identified diagnostic cutoffs for SF-CRP, they have been limited in scope and employ various CRP assay methods, without formal validation for PJI diagnosis [2,7-12,24]. This study aimed to conduct a formal single clinical laboratory validation to determine the optimal clinical decision limit of SF-CRP for the diagnosis of PJI.

## Materials and methods

This study was a retrospective analysis of deidentified data collected prospectively from 2017 to 2022. Deidentification was performed in accordance with IRB approval (WIRB-Copernicus Group Institutional Review Board (WCG IRB), Puyallup, WA, approval no. 20150222). This study was only submitted to and reviewed by Cureus.

Data collection and preparation

The data analyzed in this study were from SF samples submitted to a single Clinical Laboratory Improvements Amendments (CLIA)-certified laboratory specializing in synovial fluid analysis (CD Laboratories, Zimmer Biomet, Towson, MD), for the diagnostic testing of PJI of the hip or knee. All data were generated by laboratory instrumentation and digitally received and stored in an ACID (atomicity, consistency, isolation, durability)-compliant relational database management system with rigorous verification and error-checking procedures, for result reporting, and then de-identified for research. Sample collection was performed at over 2,600 institutions, and transport was done by an overnight courier at ambient temperature. The SF biomarker results included in testing were CRP, alpha-defensin (AD), white blood cell count (WBC), and neutrophil percentage (PMN%). Red blood cell (RBC) count, absorbance at 280 nm wavelength (A280), and the number of days between sample aspiration and sample receipt by the laboratory were included to determine specimen integrity and stability. The source healthcare institution, the source joint and laterality, and culture results were also included.

Inclusion criteria required testing for hip or knee PJI completed from 2017 to 2022; biomarker results available for SF-CRP, AD, WBC, PMN%, and culture; and specimens classified as “infected” or “not infected” using the 2018 ICM PJI Scoring System [3]. Exclusion criteria included poor specimen quality [25] (RBC > 1 million cells/µl and/or A280 outside the acceptable range of 0.342 to 1.190), specimen transport time >4 days, and specimens classified as “inconclusive” using the 2018 ICM PJI definition [3].

The 2018 ICM score included three points for either SF-WBC >3000 cells/µl or SF-AD signal to cutoff ≥1, two points for %PMN >70, and two points for a single positive culture. A score <3 was classified as “not infected”, >5 was classified as “infected”, and 3, 4, or 5 as “inconclusive” (Table 1) [3]. Scoring of the 2018 ICM definition included preoperative SF data but did not include serum data or postoperative data, as only SF data were available for this study. 

**Table 1:** 2018 International Consensus Meeting definition of PJI as applied in this study A total score >5 is classified as "infected," between 3 and 5 is classified as "inconclusive," and less than 3 is classified as "not infected." WBC: white blood cell count; µL: microliters; AD: alpha-defensin; PMN: polymorphonuclear neutrophil

Criteria	Threshold	Score
Synovial WBCs (cells/µL)	3000	3
Or		
AD signal to cutoff	1	
Synovial PMN (%)	70	2
			Single positive culture	N/A	2

CRP testing

The SF-CRP assay used was an immunoturbidity/latex agglutination assay designed for CRP measurement in serum, adapted for the quantitative determination of CRP in human SF. The assay was run on an AU680 clinical chemistry analyzer (Beckman Coulter Inc., Brea, CA) using high-sensitivity cardiac CRP (CRPH) (reagent), CRP Latex Highly Sensitive Calibrator set, and MAS liquid assayed cardiac marker controls (MAS CardioImmune XL), all sourced from Beckman Coulter, Inc., Brea, CA. Specimen viscosity was visually assessed and treated with hyaluronidase as needed on a specimen-by-specimen basis. Assay linearity from 0.9 to 82.1 mg/L for serum was confirmed to also be linear in the same range for synovial fluid. Assay accuracy was verified for SF specimens (analytical recovery 104.3%). SF-CRP testing was performed on the day of receipt of the specimens by the laboratory.

Data analysis

The data were split into a training dataset comprised of 70% of the data and a validation dataset comprised of the other 30%. While controlling for infection classification, the datasets were randomly selected across the sequential data, such that they were not temporally skewed. Using Minitab 18 (Minitab, LLC, Pennsylvania, USA), descriptive statistics were summarized for training and validation datasets, segregated based on infection classification. SF-CRP performance as a biomarker for PJI and cutoff establishment were performed using receiver operator characteristic (ROC) analysis and area under the curve (AUC) [26]. The ROC curve was plotted using sensitivity vs. 1-specificity for each CRP value in the training set in GraphPad Prism version 9.4.1 for Windows (GraphPad Software, Boston, Massachusetts USA). In Microsoft Excel (Microsoft Corporation, USA), the Youden Index (J_MAX_) was used to identify the optimum clinical decision limit from the ROC curve [27]. The cutoff was subsequently validated by calculating its sensitivity and specificity for data that were not used in its determination (validation dataset). All sensitivity and specificity values were reported with associated Clopper-Pearson 95% confidence intervals. These values were calculated in GraphPad Prism version 9.4.1 for the training data and Microsoft Excel for the validation data. Test for statistical differences of performance parameters between the training and validation datasets was performed in Minitab 18 using Fisher’s exact test.

## Results

Specimen eligibility

The initial set of 172,741 specimens was reduced to 96,061 specimens for analysis, which met all the selection parameters in accordance with the Standards for Reporting of Diagnostic Accuracy Studies (STARD) (Table 2) [28]. The primary cause for specimen exclusion was the unavailability of one or more biomarker test result(s).

**Table 2:** Data filtering and reduction via the selection criteria SF: synovial fluid; PJI: periprosthetic joint infection; SF-CRP: synovial fluid C-reactive protein; AD: alpha-defensin; WBC: white blood cell count; %PMN: percentage of neutrophils; RBC: red blood cell count; A280: optical density (i.e., absorbance) measured at 280 nanometer wavelength; µL: microliter; ICM: International Consensus Meeting

Specimen selection parameters	# of specimens
Hip and knee SF specimens submitted for PJI testing from January 2017 through May 2022	172,741
Excluded specimens	76,680
Missing one or more biomarker test results (SF-CRP, AD, WBC, %PMN, or culture) (n = 51,697)	
Poor-quality specimens (RBC > 1,000,000 cells/µL or A280 < 0.342 or A280 > 1.19) (n = 9,540)	
Transport time >4 days (n = 2,556)	
2018 ICM classification = "Inconclusive" (n = 12,887)	
Specimens for analysis	96,061

Specimen characteristics

Specimen characteristics were highly consistent across cohorts when accounting for specimen classification into the "not infected" and "infected" categories (Table 3). Analysis of the biomarker median values, culture positivity, anatomic distribution, and days from aspiration to testing reveals nearly identical specimen characteristics in both the training set and validation set. 

**Table 3:** Specimen characteristics across the training and validation sets organized into "not infected" and "infected" cohorts Continuous variables are displayed as median with the first and third quartiles. Categorical variables are displayed as a frequency and percentage. SF-CRP: synovial fluid C-reactive protein; mg/L: milligrams per liter; AD: alpha-defensin; S:CO: signal-to-cutoff ratio; WBC: white blood cells; µL: microliters; PMN: polymorphonuclear neutrophils

	Training set				Validation set			
Parameter	Not infected		Infected		Not infected		Infected	
Biomarkers	Median	Q1-Q3	Median	Q1-Q3	Median	Q1-Q3	Median	Q1-Q3
SF-CRP (mg/L)	1.0	0.4-2.4	21.0	8.6-44.7	1.0	0.4-2.4	22.0	8.8-44.9
AD (S:CO)	0.1	0.1-0.1	3.1	2.4-3.9	0.1	0.1-0.1	3.0	2.3-3.8
WBCcCount (cells/µL)	511	283-961	30,891	15,972-53,606	503	280-957	29,188	15,495-51,273
PMN (%)	34.0	24.3-47.2	94.2	90.9-96.1	34.1	24.3-47.3	94.1	90.8-96.2
Culture	Frequency	%	Frequency	%	Frequency	%	Frequency	%
Positive	277	0.5%	9,690	100%	132	0.5%	4,153	100%
Negative	57,275	99.5%	0	0%	24,534	99.5%	0	0%
Joint	Frequency	%	Frequency	%	Frequency	%	Frequency	%
Hip	4,360	7.6%	1,257	13.0%	1,915	7.8%	570	13.7%
Knee	53,162	92.4%	8,433	87.0%	22,751	92.2%	3,583	86.3%
Days from aspiration to testing	Frequency	%	Frequency	%	Frequency	%	Frequency	%
1	41,870	72.8%	6,874	70.9%	17,983	72.9%	2,893	69.7%
2	9,367	16.3%	1,668	17.2%	3,992	16.2%	742	17.9%
3	3,846	6.7%	722	7.5%	1,678	6.8%	311	7.5%
4	2,469	4.3%	426	4.4%	1,013	4.1%	207	5.0%

Training data

**Figure 1:**
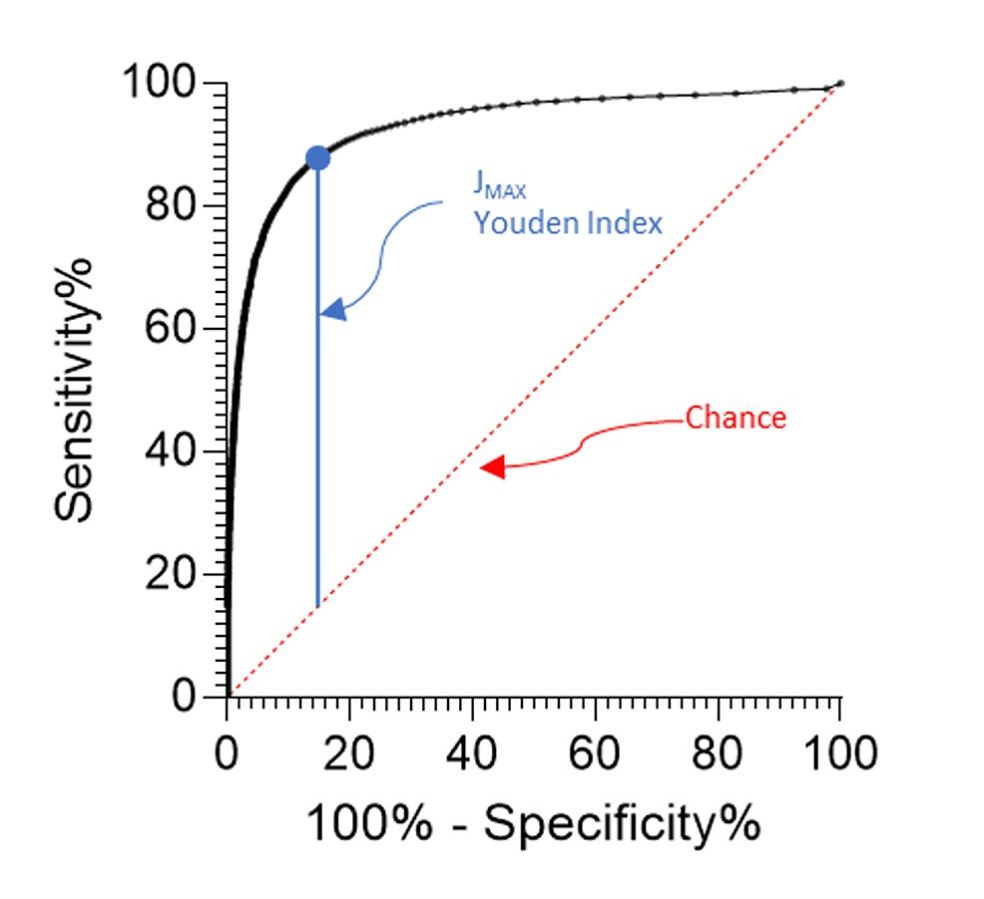
Receiver operating characteristic curve of sensitivity vs. 1-specificity for each SF-CRP value in the training set J_MAX:_ point of maximum potential discriminant effectiveness; SF-CRP: synovial fluid C-reactive protein

**Figure 2:**
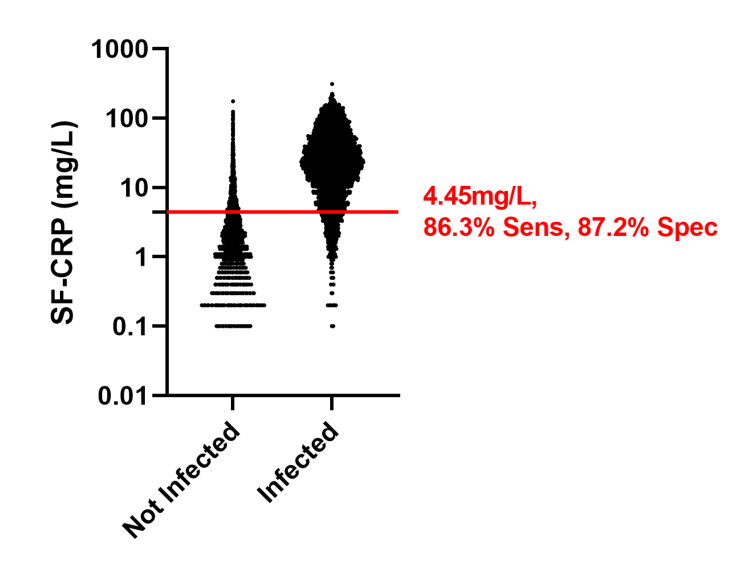
Dot plot of SF-CRP (mg/L) values for the "not infected" and "infected" cohorts of the training dataset The optimal cutoff with corresponding sensitivity/specificity is indicated. Sens: sensitivity; Spec: specificity; mg/L: milligrams per liter

In the training dataset, 57,552 specimens were classified as “not infected,” and 9,690 specimens were classified as “infected.” Sensitivity plotted against 1-specificity for each SF-CRP value in the dataset produced an ROC curve with an AUC of 0.929 (95% CI: 0.926-0.932) (Figure 1). The Youden Index, J_MAX_ = 0.735, identified the optimal value for the clinical decision limit to be 4.45 mg/L, corresponding to sensitivity of 86.3% (95% CI: 85.6-87.0) and specificity of 87.2% (95% CI: 87.0-87.4) (Figure 2).

Validation data

**Figure 3:**
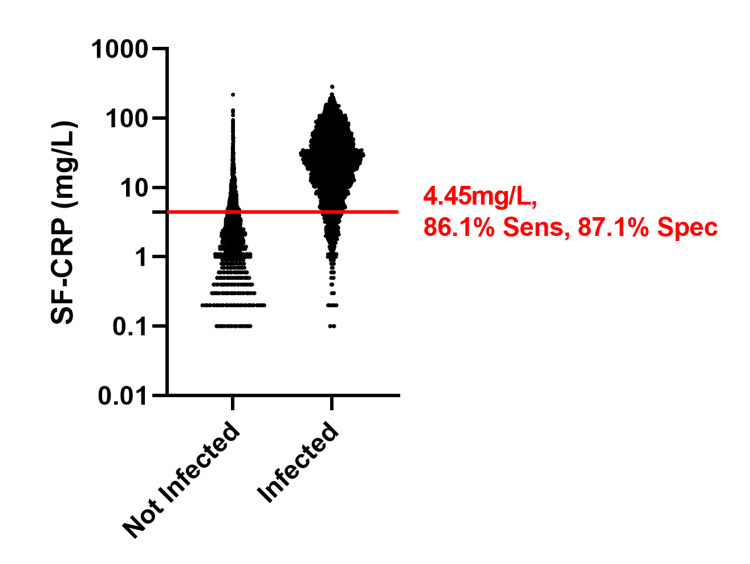
Dot plot of the SF-CRP (mg/L) value for the "not infected" and "infected" cohorts of the validation dataset Sensitivity and specificity estimated using the optimal cutoff established from the training data are indicated. Sens: sensitivity; Spec: specificity; mg/L: milligrams per liter

The optimal clinical decision limit for SF-CRP of 4.45 mg/L was then examined in the validation dataset, which included 24,666 specimens classified as “not infected” and 4,153 specimens classified as “infected.” Using the validation dataset, the optimal CRP clinical decision limit of 4.45 mg/L had a sensitivity of 86.1% (95% CI: 85.0-87.1) and a specificity of 87.1% (95% CI: 86.7-87.5) (Figure 3). No statistically significant differences were identified between the sensitivity (p = 0.767) and specificity (p = 0.699) results from the training and validation data.

## Discussion

In this study, we aimed to assess the diagnostic performance of SF-CRP for detecting PJI using a training dataset consisting of 67,242 specimens and an independent validation set of 28,819 specimens not included in the training data. Our dataset greatly surpassed the scale of previous studies [2,7-12,24] and employed a consistent SF-CRP assay across all samples. While the clinical laboratory in this study had previously validated the SF-CRP assay in combination with other biomarkers [23], this study represents, to our knowledge, the largest single-laboratory formal validation of SF-CRP with a clinical decision limit for PJI.

In this study, SF-CRP exhibited a good diagnostic performance for PJI, with an AUC of 0.929, a sensitivity of 86.1%, and a specificity of 87.1%. We determined the clinical decision limit for PJI positivity to be 4.45 mg/L, employing Youden Index optimization for sensitivity and specificity pairs. The objective of employing both training and validation samples was to demonstrate the robustness of the clinical decision limit and its associated sensitivity and specificity in a new set of samples, which were not involved in setting the clinical decision limit. Comparing the diagnostic performance of the SF-CRP assay between the test and validation sample sets showed no statistically significant differences, affirming the validity of the 4.45 mg/L clinical decision limit beyond the original training set.

Reviewing the existing literature on SF-CRP for PJI diagnosis, we identified 17 publications since 2012. These studies presented a wide spectrum of assay methodologies without standardization across laboratories (Table 4). Furthermore, most of these studies had limited sample sizes, resulting in SF-CRP cutoffs ranging from 1.6 to 12.9 mg/L for optimal PJI detection (Table 4). This variability in PJI-optimized SF-CRP thresholds likely arose from differences in CRP assay methodologies and the small sample sizes that limit the precision of determining the clinical decision limit. Nonetheless, a review of this literature revealed a strong correlation between serum and SF-CRP [7,8,11,24], with SF-CRP generally demonstrating superior diagnostic performance [7-12]. Our study's clinical decision limit of 4.45 mg/L falls within the mid-range of existing literature values and is based on a dataset over 100 times larger than the largest previous study involving 621 patients [7]. Therefore, our study provides robust confirmation of the existing literature on SF-CRP for PJI diagnosis. Moreover, our study's findings closely align with those of a meta-analysis that reported a sensitivity and specificity of 85% and 88%, respectively, for SF-CRP in the existing literature [29].

**Table 4:** Literature reporting an SF-CRP cut-off for PJI POC: point of care; nr: not reported; CI: confidence interval; mg/L: milligrams per liter; HKS: hip/knee/shoulder * The method of cut-off determination was random forest analysis; all others used receiver operating characteristics (ROC) curves, most with Youden Index. ** The upper 95% CI value appears to have a typographical error in the publication.

Study	Date (mmm-yy)	1st Author	Sub-group	# of Cases	Cut-off (mg/L)	Sensitivity (%)	95% CI	Specificity (%)	95% CI
Current	Dec-23	Miamidian	N/A	28,819	4.45	86.1	85.0, 87.1	87.1	86.7, 87.5
1	Nov-23	Grzelecki [15]	lab	145	9.6	72.7	57.2, 85.0	98.7	92.9, 100
			lab	145	2.7	90.9	78.3, 97.5	94.7	87.1, 98.6
			POC1	145	3	68.2	52.4, 81.4	77.6	66.6, 86.4
			POC2	145	8	90.9	78.3, 97.5	90.8	81.9, 96.2
			POC3	145	10	77.3	62.2, 88.5	94.7	87.198.6
			POC4	145	10	77.3	62.2, 88.5	96.1	88.9, 99.2
2	Mar-23	Diniz [16]	N/A	102	2.7	84.4	nr	92.1	nr
3	Jun-22	Baker [7]	N/A	621	6.9*	74.2	nr	98	nr
4	May-22	Felstead [17]	N/A	161	10	88.24	76.13, 5.56**	90.91	83.92, 95.55
5	Dec-21	Yu [8]	N/A	139	1.632	93.6	nr	68.8	nr
6	Aug-21	Wang [10]	N/A	97	7.26	84.62	69.5, 94.1	93.1	83.3, 98.1
7	Jun-21	Praz [13]	N/A	196	4.4	82.5	70.7, 94.3	88.3 95	82.3, 94.3
				196	2.7	85	70.9, 92.9	76.6 95	67.9, 83.5
8	Jun-20	Sharma [18]	N/A	107	5.65	80	nr	92	nr
9	Apr-19	Plate [14]	shoulder	21	2.4	100	nr	78	nr
			knee	91	12.9	98	nr	71	nr
			hip	80	2.9	93	nr	86	nr
			all HKS	192	2.9	88	nr	82	nr
10	Sep-18	Tahta [21]	N/A	38	11.7	76.4	62, 97	90.4	80, 100
11	Jun-18	Gallo [19]	N/A	240	8.8	91.7	73.0, 98.9	100	94.5, 100
12	Mar-17	Sousa [20]	N/A	55	6.7	78.3	nr	93.8	nr
13	Nov-14	Deirmengian [22]	N/A	95	12	90	73, 98	97	90,100
14	Sep-14	Deirmengian [23]	N/A	149	3	97.3	85.8, 99.6	78.6	69.8, 85.8
15	Jul-14	Tetreault [11]	N/A	119	6.6	88	82, 93	85	79, 91
16	Sep-12	Parvizi [24]	N/A	63	9.5	85	nr	95	nr
17	Jan-12	Parvizi [12]	N/A	66	3.7	84	nr	97.1	nr

In addition to its likely superior diagnostic performance [7-12], there are qualitative reasons supporting the preference for a validated SF-CRP test over a serum CRP test for diagnosing PJI. First, the SF-CRP test as described in this study is optimized and validated for PJI diagnosis, offering consistent units of measure and thresholds for all patients, simplifying interpretation for clinicians. By contrast, serum CRP tests provided by various laboratories lack optimization for PJI and involve varying units of measure and thresholds, potentially leading to misinterpretation or errors [30]. Second, as SF-CRP and other SF tests for PJI are drawn from the same SF sample, they provide a contemporaneous snapshot of the disease state, eliminating temporal concerns. Serum CRP, on the other hand, is drawn days to weeks apart from SF analysis, introducing temporal sampling variation that may not accurately reflect the patient's disease progression or state. Therefore, if SF-CRP and serum CRP exhibited similar diagnostic performance for PJI, a validated SF-CRP would be qualitatively preferable in the clinical setting.

This study not only corroborates existing literature on SF-CRP's diagnostic performance but also sets a laboratory benchmark for clinically validating the SF-CRP test for PJI. It is important to recognize the distinction between generic tests and disease-specific, validated tests in the realm of diagnostic laboratory testing. Existing generic laboratory tests for serum CRP and even SF-CRP lack optimization and validation for diagnosing PJI, yielding varying units of measure and diagnostic thresholds not intended for PJI diagnosis, possibly conflicting with existing guidelines [30]. Therefore, clinicians relying on these generic tests may face uncertainty regarding the applicability of laboratory results to patients being evaluated for PJI. To effectively utilize a SF-CRP test for PJI diagnosis, clinicians should consider if the laboratory conducting the test has undertaken proper validation to establish an optimal clinical decision threshold, ensuring the interpretability of test results for PJI diagnosis.

While this study offers valuable insights, it has several limitations. First, it relied solely on SF laboratory data and lacked access to clinical data, such as serology and tissue cultures. All sample classifications as infected or not infected were based solely on SF data. The 2018 ICM definition of PJI explicitly states that its criteria can be applied using only preoperative data and clarifies that all tests are not required for completing the definition [3]. Nevertheless, we want to emphasize that we did not employ serology or tissue culture when classifying samples. Our results align closely with the existing literature, mitigating concerns that this may affect the study's outcomes. Second, this is a retrospective study and cannot achieve the stringent patient selection and clinical data analysis possible in prospective studies. Nonetheless, we believe that collecting data directly from laboratory instruments in a prospective manner enhances data integrity, minimizing the potential for human error in data collection.

## Conclusions

In summary, for the specific SF-CRP assay methodology employed at one clinical laboratory for PJI diagnostic workup, we determined the optimal clinical decision limit to be 4.45 mg/L, validated with sensitivity and specificity values of 86% and 87%, respectively. This information is pivotal for conducting a reliable SF-CRP assay. However, it is important to note that this clinical decision limit is not applicable to other assay methodologies or laboratories.

Published literature suggests that SF-CRP may match or surpass serum CRP in diagnosing PJI. Employing a CRP assay with a validated methodology and laboratory-specific cutoff for synovial fluid allows contemporaneous measurements for all biomarkers required in multi-criteria PJI diagnostic definitions, potentially enhancing clinical accuracy.
